# Measuring and shaping the nutritional environment via food sales logs: case studies of campus-wide food choice and a call to action

**DOI:** 10.3389/fnut.2024.1231070

**Published:** 2024-06-04

**Authors:** Kristina Gligorić, Robin Zbinden, Arnaud Chiolero, Emre Kıcıman, Ryen W. White, Eric Horvitz, Robert West

**Affiliations:** ^1^Data Science Lab, EPFL, Lausanne, Switzerland; ^2^Population Health Laboratory (#PopHealthLab), University of Fribourg, Fribourg, Switzerland; ^3^School of Population and Global Health, McGill University, Montreal, QC, Canada; ^4^Swiss School of Public Health (SSPH+), Zurich, Switzerland; ^5^Microsoft Research, Redmond, WA, United States; ^6^Office of the Chief Scientific Officer, Microsoft, Redmond, WA, United States

**Keywords:** food choice, measurement, monitoring, determinants, digital traces, health, sustainability, policy

## Abstract

Although diets influence health and the environment, measuring and changing nutrition is challenging. Traditional measurement methods face challenges, and designing and conducting behavior-changing interventions is conceptually and logistically complicated. Situated local communities such as university campuses offer unique opportunities to shape the nutritional environment and promote health and sustainability. The present study investigates how passively sensed food purchase logs typically collected as part of regular business operations can be used to monitor and measure on-campus food consumption and understand food choice determinants. First, based on 38 million sales logs collected on a large university campus over eight years, we perform statistical analyses to quantify spatio-temporal determinants of food choice and characterize harmful patterns in dietary behaviors, in a case study of food purchasing at EPFL campus. We identify spatial proximity, food item pairing, and academic schedules (yearly and daily) as important determinants driving the on-campus food choice. The case studies demonstrate the potential of food sales logs for measuring nutrition and highlight the breadth and depth of future possibilities to study individual food-choice determinants. We describe how these insights provide an opportunity for stakeholders, such as campus offices responsible for managing food services, to shape the nutritional environment and improve health and sustainability by designing policies and behavioral interventions. Finally, based on the insights derived through the case study of food purchases at EPFL campus, we identify five future opportunities and offer a call to action for the nutrition research community to contribute to ensuring the health and sustainability of on-campus populations—the very communities to which many researchers belong.

## 1 Introduction

The concept of a modern campus evolved to be more than a collection of buildings and grounds that belong to an institution. A modern campus is a complete local community. In such a situated context, people spend significant parts of their time working, educating, or being educated, but also socializing, learning, doing sports, or entertaining themselves. People also consume food regularly and globally, in university, corporate, medical, industrial, and other types of campuses. The food consumed on campuses has broad implications for the people on campus and the general population, impacting health and the environment (1, 2). Given the need to actively address the challenges of climate change (3), stakeholders have a growing interest in reducing their campuses' environmental impact.

Social issues, including health and environmental issues, as well as situated socialized contexts such as campus environments, have been areas of interest in nutrition research. The nutrition research community is well-positioned to help campus communities, universities, corporate stakeholders, and policymakers promote these values. To be able to take action toward the goals of ensuring on-campus health and sustainability, it is important to know, to begin with, what foods are consumed on campus and in what context.

However, answering this question poses two major challenges. First, it is challenging to provide good measurements. Traditional methods such as surveys rely on self-reporting, face limitations such as under-reporting of undesired behaviors (4–6) and cannot capture temporal dynamics accurately. Researchers have begun to explore digital traces, e.g., from social media (7), search engines (8–10), or food-tracking applications (11, 12), however, it remains unclear to what extent such distant proxies reflect real food consumption (13–15). Second, measurements from a single campus do not necessarily generalize to other campuses since behaviors are observed at different times, locations, and food landscapes. The worldwide campuses are fundamentally incomparable isolated eco-systems. Hence, much about fundamental campus dietary behaviors with implications for health and the environment—such as meat vs. meat-free meal consumption and consumption of caffeinated or alcoholic drinks—remains unknown. New measurement methods are needed to overcome these challenges and collect dynamic, fine-grained, and generalizable data about the diet of campus populations.

In the present work, we investigate how passively sensed^1^ food purchase logs can be used to shape the nutritional environment, and argue that nutrition researchers are well-positioned to contribute to improving the nutritional environment at their institutions. To that end, we introduce case studies of an anonymized dataset of passively collected logs of food purchases made on a large university campus and perform statistical analyses of purchasing behaviors with policy implications. The present work studies purchasing behaviors on a university campus, however, the notion of campus, the insights, opportunities, and challenges refer to similar, more or less closed environments.

In this work, we present the following contributions. First, through a case study of purchases at one campus, we report quantitative empirical analyses of passively sensed food purchases. In doing so, we aim to characterize the determinants of food consumption and demonstrate how studies relying on passively sensed purchase logs have the potential to support the stakeholders in their efforts to understand food choice and consumption. We demonstrate the utility of measurement and monitoring via large-scale digital traces through case studies leveraging real-world passively collected food sales logs. In particular, aiming to understand why people on campus purchase the foods they do, we identify spatial proximity, food item pairing, and academic schedules (yearly and daily) as important determinants.

We then formulate a set of further research questions that showcase the breadth of insights that can be sourced from the purchase logs, beyond the specific campus studied here. We aim to make a case for re-purposing such data, which are often available by default and can serve as a valuable source of information to harness in campus environments to monitor and measure nutrition. Finally, we conclude by offering future opportunities and a call to action for the nutrition research community to contribute to ensuring the health and sustainability of the institutions researchers are part of. We argue that it is crucial for researchers to understand the values embedded in the existing food systems at campuses, the existing data collection and analysis practices, and the values and priorities of the individuals impacted by the food offering and food consumption.

## 2 Materials and methods

### 2.1 Dataset: food purchase logs

We leverage an anonymized complete dataset of food purchases made on the École Polytechnique Fédérale de Lausanne (EPFL) university campus. The data spans eight years, from 2010 to 2018, and contains about 38 million transactions, of which about 18 million were made with a badge that allows linking to an anonymized person's ID.

The statistical analyses are based on the seven-year period from Jan 1 2012 to Dec 31 2018 with menu data available. The data includes 38.7 k users, who, on median, are observed for a time period spanning 578 days and make 188 transactions. The analyses include all the transactions within that period, unless otherwise noted. Each transaction is attributed with the time it took place, information about the sale location, the purchased items, their quantity, and price. Sold food items are mainly ready to consume and are associated with unstructured textual descriptions (e.g., “coffee”, “croissant”, “Coca-Cola can”).

The data covers all the food outlets permanently located on campus, including restaurants, cafes, and vending machines. During the entirety of the studied period, there were twelve major catered shops located throughout the campus,^2^ and a number of self-service vending machines. The shops typically open Monday through Friday, opening at 07:00 and closing at 20:00, while vending machines are available 24/7. The shops generally are not open during the weekend. All shops offer lunch, while breakfast and dinner offerings vary across shops. Note that student halls in the vicinity of the campus do not have associated dining halls. Hence, the catered shops are the main campus-provided food option for students. In the close proximity of the campus, there are several non-affiliated food providers, including two seated restaurants (an Italian restaurant and a fast food outlet), two grocery stores, a cafe, and temporarily located food trucks.

In addition to anonymized on-campus purchase logs, we also analyze a smaller-size enriched dataset gathered during a three-week campus-wide sustainability challenge in November 2018, during which 1,031 consenting and volunteering participants formed 278 teams to compete in taking sustainable actions (e.g., taking the stairs instead of the elevator, or consuming a vegetarian meal). For this subset of individuals, we leverage demographic information and identify all of their transactions present in the purchase logs. This results in a subset of 585,812 transactions annotated with demographic information.

The unstructured food item textual descriptions were additionally mapped to categorical labels such as “sandwich” or “dessert” by a research assistant who labeled the 500 most frequently purchased items, which account for 95.4% of the total volume of item purchases observed in the dataset. Further supplementary information about the dataset are outlined in the Supplementary material.

### 2.2 Ethical considerations

Nutrition is a potentially sensitive personal behavior. To protect user privacy, the log data used here was accessed exclusively by the personnel involved in this project, and stored and processed exclusively on internal servers. The data was obtained with approval from Data Protection Officer^3^ and was anonymized before it was made available to the researchers for analysis.

In the data collection and analysis process, we worked directly with campus food-providing administration and transaction system managers who exported and anonymized the data, to understand the information about the food items and restaurants encoded in the dataset. Finally, we note that our work leveraging purchase logs was conducted retrospectively on data that had been collected passively in order to support campus operations. Thus, our analysis did not influence users in any way. Purchases executed by sustainability challenge participants were analyzed retrospectively as well, without influencing them in any way.

## 3 Results

Motivated by the existing knowledge about human dietary behaviors, we perform descriptive statistical analyses of food-purchasing behaviors in order to characterize prominent on-campus food choice determinants. Since human dietary behaviors and choices are known to exhibit spatio-temporal regularity (16) and recurrent routine-like patterns (17, 18), we analyze dimensions along which purchasing behaviors vary, including temporal, spatial, and choice regularities (Section 3.1). We then analyze the known issues that campus food consumption faces, including ensuring sustainability and health, and tackling the impacts of regular campus operations (Section 3.2). In what follows, we describe our findings and discuss how these insights can inform on-campus policy-making.

### 3.1 Spatio-temporal and choice determinants of food purchases

#### 3.1.1 Temporal determinants: the heartbeat of the campus

**Figure 1:**
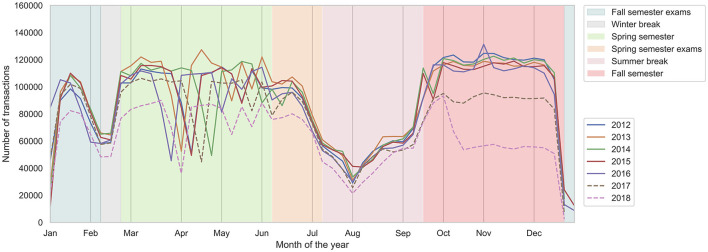
Across the month of the year (on the x-axis), the number of executed transactions (on the y-axis), across the years. Background color marks the yearly academic schedule. Note that in 2017 and 2018, the number of transactions during the semester decreased (dashed lines), likely due to the opening of shops close to campus that do not support purchases with the identifying badge.

**Figure 2:**
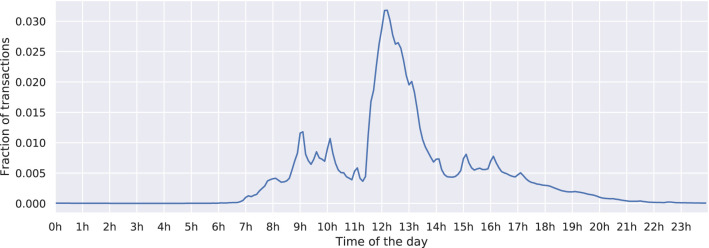
The histogram of the transactions by the time of the day. The shops typically open at 07:00 and close at 20:00, while vending machines are available 24/7.

During a year, the academic calendar dictates life on campus. The three important periods in the academic calendar of the studied campus—semesters, exam sessions, and breaks—are marked in Figure 1. We find that the number of transactions peaks during the spring and fall semesters when students are attending lectures, drops during the exam sessions, and reaches the minimum during the winter and summer breaks. During the course of a day (Figure 2), the transactions peak in the morning, during the time of lunch, and during the afternoon or evening snack time.

#### 3.1.2 Spatial determinants: migration patterns between shops

**Figure 3:**
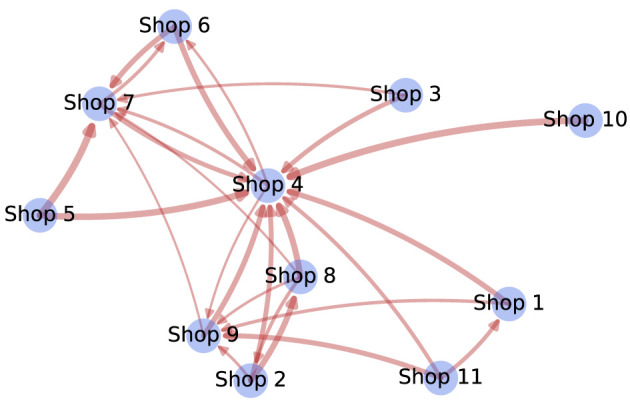
The directed weighted graph representing association rules between different shops. Nodes represent shops, and the edges represent association rules. The edge thickness is proportional to the confidence of the rule.

After temporal, we describe spatial determinants in the purchasing behaviors. We are interested in understanding the regularities in visits to shops. To that end, we perform an association rule analysis. Across all individuals, we consider the shops where a given individual has executed transactions during a week. We then apply the Apriori algorithm (19), an algorithm for the discovery of association rules between shops. Association rules describe regularities between items in transaction data. For example, a rule {X} → {Y} found in the transaction logs would indicate that if a person visited shop *X*, they are likely to also visit shop *Y* during the same week. In Figure 3, the graph depicts the confidence of the association rules found using the Apriori algorithm, defined as the percentage of all transactions satisfying *X* that also satisfy *Y*. This approach lets us monitor shop migration patterns (Figure 3). Rules with confidence greater than the 0.2 threshold are displayed with an edge.

For instance, a thick arrow from node *Shop 10* to node *Shop 4* means that there is a high probability that an individual who went to *Shop 10* also went to *Shop 4* during the same week (shop names are anonymized). *Shop 9* is the on-campus bar that also serves food, while the other shops are on-campus cafeterias that serve meals, beverages, and snacks.

Overall, we find that the distribution of the edges is linked to the geographic locations of the shops. For instance, *Shop 4* is the central place in the graph as there are many arrows with high confidence coming to it, and this cafeteria is indeed at the geographical center of the campus. Moreover, the nearby cafeterias on the campus, frequently visited by students, form a cluster in the shop co-occurrence graph. *Shop 8, Shop 11, Shop 1, Shop 9* and *Shop 2* are near-by cafeterias on the campus, frequently visited by students, and we observe that they form a cluster in the shop co-occurrence graph.

#### 3.1.3 Food-choice regularities and routines: co-purchasing patterns

**Figure 4:**
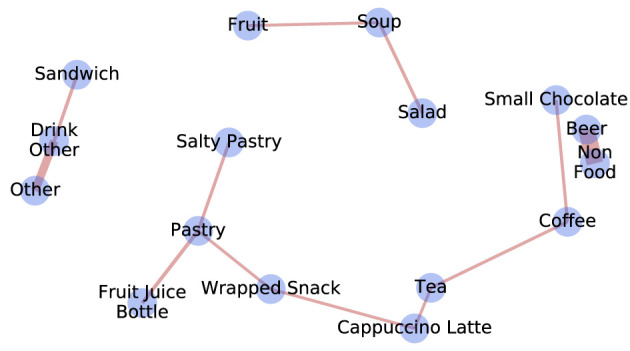
The weighted graph representing association rules between different food categories. Nodes represent food categories and the edges represent rules. The edge thickness is proportional to the lift of the rule.

Next, we study the regularities in purchases of specific foods, performing association analysis, as explained above. We consider purchases composed of food items belonging to different categories, ı.e., for each purchase, the food categories a person has purchased. In Figure 4, we show the lifts of the association rules found using the Apriori algorithm, defined as the ratio of how frequently the rule appears in the dataset to that expected if *X* and *Y* were independent. Note that the lift is a symmetric measure; therefore, the graph is not directed. We obtain a graph where neighbor nodes are food categories that tend to be purchased together, e.g., *Salty Pastry* and *Pastry*. *Soup, fruit*, and *salad* are often co-purchased. Note that the *Non-Food* category contains only products linked to beer, such as glasses on deposit at the on-campus bar.

#### 3.1.4 Implications for policies

To summarize, we reveal regularities in how persons on-campus transition between cafeterias during a week and how food items belonging to different food categories are combined. Since the transitions between cafeterias are governed by geographical proximity (Section 3.1), our analyses imply that the decisions about offering at cafeterias should not be taken in isolation, but should account for such spatial migration patterns. Similarly (cf. Section 3.1), the offer should take into account the frequent item pairings because it is not enough to consider foods in isolation (e.g., a sandwich can have good nutritional properties, but if often purchased together with a beverage with high sugar content, promoting it might not be optimal). Analyses of purchase logs can detect these issues and inform measures taken to improve the experiences around food on campus.

### 3.2 Specific dietary behaviors: sustainability, health, and the academic schedule

#### 3.2.1 Food sustainability: vegetarian meals

We now turn to analyze specific dietary behaviors relevant to sustainability and health on campus. We first analyze vegetarian meal purchases since the consumption and production of meat have a negative impact on the environment (20). Considering the frequency at which vegetarian meals are purchased out of all purchased meals, we find that, on average, across individuals, 5.3% of purchased meals are vegetarian. Vegetarian meals are the most popular among Ph.D. students (11.2%), followed by undergraduate and master students (8.3%), staff (5.9%), and other statuses (e.g., interns and visitors; 2.4%). Vegetarian meals are the most popular among 21–30-year-olds vs. other age groups (16– 20-year-olds and over 30-year-old) and among women vs. men (8.5% vs. 7.2%). We also note that the proportion of vegetarian meals is monotonically increasing over time, rising from less than 3.98% in 2012 to close to 7.96% in 2018. This rise is likely due to the rise in awareness regarding the effect of meat production and consumption on the environment and health, but also due to the university adapting its offering to cater to these trends. The complete distribution is presented in the Supplementary material.

#### 3.2.2 Potentially harmful dietary behaviors

We next analyze purchases of four types of products that could potentially imply harmful effects on health, albeit to a varying extent depending on the number of daily servings and the specific context (including specific nutritional values, ingredients, and ways of preparation, and added sugar content). We focus on (1) beer, (2) energy drinks, (3) coffee, and (4) vending machine items. We measure the fraction of transactions including such products, out of all purchased products, across subpopulations of status, gender, and age.

First, regarding beers, we find that, on average, across individuals, 2.9% of transactions contain a beer. Beer purchases are the most prevalent among “other” statuses, students, Ph.D. students, 21-to-30-year-olds, and men (3.7% men vs. 1.9% women). Monitoring beer purchases is important since consumption of alcoholic beverages in excessive amounts is not recommended (21). Second, on average, across individuals, 0.15% of transactions contain an energy drink. Energy drinks are the most prevalent among students and Ph.D. students, 26-to-30-year-olds, and men (0.1% men vs. 0.041% women). Monitoring energy drinks is pressing since excessive consumption of caffeinated energy drinks have been reported in association with adverse health effects (22). Third, monitoring coffee purchases, on average, across individuals, we find that 15.4% of transactions contain a coffee. Coffee is the most prevalent among women, staff members, and older subpopulations (31-year-olds and older). We note that the question of the effects of drinking coffee on health is nuanced and multifaceted (23). Lastly, monitoring vending machine purchases, on average, across individuals, we find that 6.2% of transactions contain a vending machine item. Vending machine items are the most prevalent among students and 21 to 25-year-olds. Food products available in vending machines often have a high amount of sugar, and vending machines tend to be nutritionally poor (24), highlighting the importance of minitoring such purchases. The complete distribution is presented in the Supplementary material.

#### 3.2.3 Implications for policies

We observe significant differences between subpopulations. Overall, we find that purchases reflecting potentially harmful dietary behaviors are relatively prevalent, especially vending machine purchases (overall, 6.2% or 1 in 16 transactions a person makes contains a vending machine item). Students, Ph.D. students, younger subpopulations, and men are the most susceptible to purchasing potentially harmful items, while vegetarian meals are most popular among Ph.D. students, people between 21 and 30 years of age, and women. These insights can help stakeholders design targeted interventions and campaigns. For instance, campaigns aiming to promote purchases of healthier alternatives might be located and phrased such that they are geared toward students and younger subpopulations who are the most susceptible to purchasing potentially harmful items. Communicating attractiveness and health of food options through tailored advertisements (25), visual cues (26), and plate graphics (27) might be effective too, as can sustainability challenges. Similarly, interventions providing discounts on healthy food items outside of regular opening hours could be effective in reducing purchases of potentially unhealthy items. Finally, more research is needed to systematically identify social gatherings and events on campus that increase the risk of repeated unhealthy behaviors, and design interventions to address them.

#### 3.2.4 Yearly and daily academic schedules determine on-campus food choice

**Figure 5:**
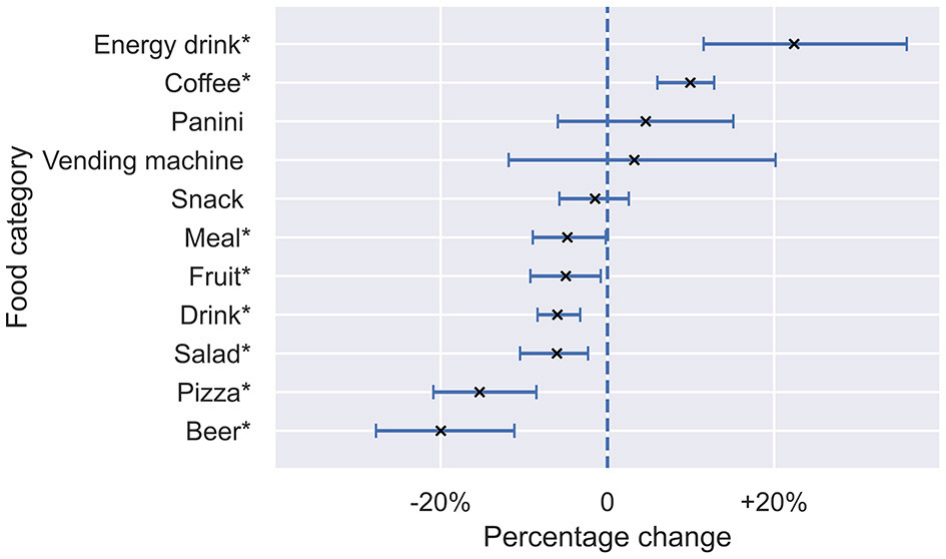
For different food categories (on the y-axis), the percentage change (on the x-axis) in the fraction of all purchases that contain the food item during exam weeks, compared to lecture weeks. Positive values mark categories with increased purchases during exam weeks and negative values mark categories with decreased purchases during exam weeks, compared to lecture weeks. Error-bars mark bootstrapped 95% confidence intervals obtained by resampling respective weeks. Stars mark food categories with percentage change significantly different from zero.

Finally, we aim to understand how campus operations determine purchasing behaviors. First, to understand how purchasing behavior changes during the exam session, we compare the differences in purchases between semesters and exam sessions. Monitoring weeks of the entire studied period, in Figure 5, we find that, during the exam weeks, compared to semester weeks, there is a significant increase in the relative frequency of purchases of energy drinks (+22.4%), coffee (+9.9%), and a decrease in the relative purchasing frequency of beer (-20.0%), pizza (-15.3%), drinks (-6.0%), and fruit (-5.0%). For instance, the fraction of purchased energy drinks, which has the greatest change between exam weeks and semester weeks, peaks during the fall semester exams (peak occurs on the last week of the year when 1% or 1 in 100 purchased items is an energy drink). As expected, these effects are stronger among students than among staff members, who are less affected by the academic calendar (Supplementary Figures S5, S6).

**Figure 6:**
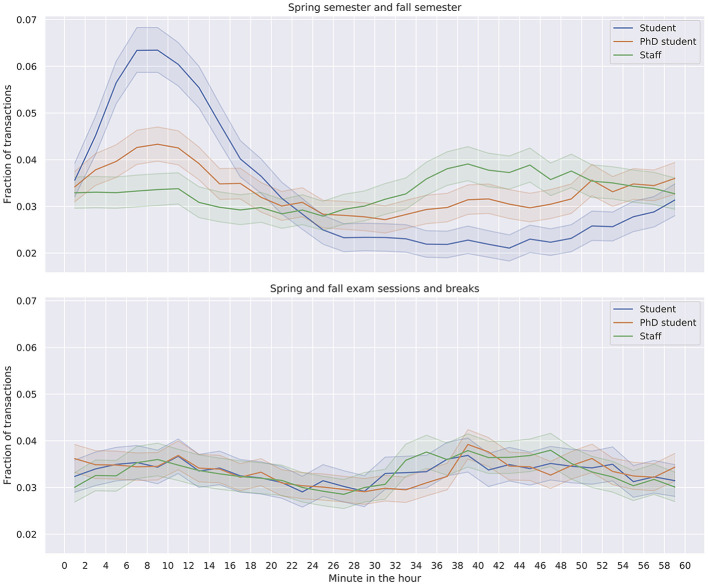
By the minutes of the hour (on the x-axis), the fraction of executed transactions during the minute, separately during the semesters **(top)** vs. during the exams sessions and breaks **(bottom)**, and separately by status. Error-bars mark bootstrapped 95% confidence intervals.

During the fall and spring semesters, each class begins after the first quarter of each hour and ends at the end of the hour, with a break of 15 minutes between two classes. Investigating the impact of the academic calendar on purchasing behaviors within an hour (Figure 6), we observe different behavior depending on the individual's status and whether the transactions are made during the semesters or not. During the spring and fall semesters, students' transactions peek at the 8th minute in the hour, during the 15-minute break, and consequently drop (Figure 6, top). Transactions executed by the staff members do not exhibit such a pattern. Ph.D. students are in between staff and students. These differences between staff members and students disappear during the exam sessions and breaks when there are no 15-min breaks (Figure 6, bottom), implying that hourly patterns are indeed linked with the academic calendar since students tend to take advantage of the 15-min break to buy drink or food.

#### 3.2.5 Implications for policies

To summarize, we find that academic schedules determine food consumption on campus, both at the yearly level (lecture season vs. exam season) and the daily level (lectures vs. breaks). On the yearly level, exam sessions are associated with surges in the consumption of both coffee and energy drinks (Figure 5), while on a daily level, the 15-min break between lectures drives food consumption, particularly among students and during lecture weeks. Exams are associated with increases in purchases of potentially unhealthy products, likely due to stress and performance desires. These insights highlight the need for policy-makers to do more to promote student well-being as stress and anxiety levels are elevated among university and college students (28, 29). Modifying the food offer and making exam sessions a better experience for students by encouraging socialization might be promising directions, as socialization might be reduced during exams since beer and pizza purchases decrease, cf. Figure 5. Such community-setting food environment interventions targeting young adults are typically associated with improvements in diets and nutrition outcomes (30). Similarly, insights regarding the 15-min break imply that purchase line congestion might occur. Interventions mitigating the surge in purchases and modifying the offering might be effective, for instance, by opening additional dedicated checkouts with modified healthy snack offerings.

## 4 Discussion

### 4.1 Summary of main findings

The presented results imply that approaches leveraging passively sensed anonymized data should be an essential component in efforts to monitor the evolution of food consumption on campus while being aware of complex spatio-temporal determinants and differences between subpopulations. For instance, the finding that the academic schedules determine food consumption on the studied campus at the yearly and daily level, and the fact that exams are associated with significant increases in purchases of potentially unhealthy products, highlight the need for policy-makers to do more to promote student well-being.

These findings have direct implications for developing new methods for population nutrition monitoring, encouraging better eating practices, and optimizing food offerings. By capturing nearly all on-campus food consumption, the purchase log analysis approach complements survey-based methodologies, which likely under-report (5, 6) stigmatized consumption of unhealthy items.

#### 4.1.1 Limitations

Certain limitations should be kept in mind when interpreting our results. First, the presented case study is limited to a single campus. Behaviors on the studied campus can fundamentally differ from behaviors on other campuses. External validity and generalization are not guaranteed. Future work should determine to what extent behaviors measured on campus reflect behaviors in other on-campus settings, which might differ in their location, climate, the characteristics of the population, and other environmental properties. We also note that self-selected sustainability challenge participants are not a representative sample of the entire campus population (Supplementary material, Section 1.2). Second, on the studied campus, the unstructured food item labels and the information derived from them are incomplete. For instance, cash transactions cannot be mapped to individuals (46.92% of transactions could be mapped to a specific user). Further information about the difference between the entire dataset, non-identifiable cash or card transactions, and identifiable transactions executed with the badge are listed in Supplementary Table S1. Overall the distributions are similar across the datasets, although systematic differences exist. Similarly, he vegetarian tag was deduced from the name and type of the product and is, therefore, not necessarily always correct. Third, the log data does not directly capture food consumption but provides indirect proxies via purchasing. We also note that the described analyses do not necessarily establish causality. It is reasonable to assume that students and staff indeed consume the food that they purchase. However, one cannot eliminate the possibility of persons borrowing the card, or paying for items consumed by other people.

Future work should determine to what extent behaviors measured on campus reflect consumption of food purchased outside of the campus and off-campus behaviors. For example, people who primarily eat home-cooked or delivered food might present a skewed representation of their diet in the studied cafeteria purchase logs, and the fact that individuals might consume delivered or home-cooked food introduces unobserved variables into our analyses. Meals consumed at restaurants nearby but outside of campus are similarly not captured, while opening and closing of shops close to campus can impact on-campus behaviors. Furthermore, cultural factors and dietary preferences might play a role in determining whether students and staff choose to rely on food options provided on campus to begin with (31, 32). Related to cultural background, we note that the number of unique users in the dataset grew as the staff and student body expanded (by around 40%, from 12.5 k unique users in 2012 to 17.5 k unique users in 2018). This increase coincides with a recorded growth in the international student population (33), highlighting the importance of campus stakeholders adapting food offerings to various cultures and cuisines.

#### 4.1.2 Further research questions

The insights from the statistical analyses inform a wide range of research questions that can be addressed leveraging anonymized passively sensed purchase logs, potentially in conjunction with other data sources. For instance, future work should understand how food choice is determined by the availability of options, and how the lack of food offerings at late hours might be linked with potentially unhealthy patterns, including vending machine purchases. How does food choice evolve over the course of a day and a week? Do people tend to consume healthier foods at the beginning of the day or the week? To what extent is food choice determined by geographical proximity to options? How much work would people be willing to put in to reach better options? How does the opening of unaffiliated premises near campus affect on-campus meal consumption? Similarly, how does weather affect food purchases? Could random variation in weather be leveraged to study spatial proximity? Additional opportunities to tackle potentially harmful dietary behaviors include understanding their onset. Can on-campus alcohol drinking and energy drink consumption be predicted? How does eating with others impact on-campus food choice, and can social challenges lead to more sustainable on-campus behavior?

#### 4.1.3 Further background and scope

We now turn to situate our findings with respect to the existing literature on monitoring and measuring nutrition with digital traces in a campus-wide setting. A rich body of previous work examined the factors that represent barriers and enablers to healthy eating in campus environments (34). The dominant factors include price, value for money, healthfulness, and taste (35, 36). Previous work has studied the specific, potentially harmful, dietary behaviors in on-campus setting, including the consumption of energy drinks (37, 38), alcohol (39, 40), vending machine foods (24, 41), and a failure to reach the nutritional recommendations (42). On-campus dietary interventions that aim to improve food availability, accessibility, prices, and promotions through policies received considerable attention from researchers (35, 43–45). Accessibility and price are important factors on the studied campus, too. For instance, during the studied period, the average price of a purchased meal was monotonically increasing, and grew by 30.31% between 2012 to 2019 (from 7.62CHF to 9.93CHF). Similarly, the price of a non-meal item grew from 2.54CHF in 2012 to 3.85CHF in 2019, further highlighting the role of affordability of both on- and off-campus options.

However, understanding all the determinants of food consumption and identifying intervention effectiveness is challenging since the key factors, such as availability and attitudes, change over time, interact, and do not necessarily generalize across campuses. Moving forward, there is a need for unified frameworks to better monitor and understand food consumption in campus environments worldwide.

Studying diets through digital traces has been an active area of research (10, 46, 47). Large-scale passively sensed signals have been harnessed in university campus environments to measure factors of well-being (29) and performance (48), pointing toward the feasibility of leveraging behavioral traces for campus-centric applications (49–51).

Nonetheless, while large-scale digital traces are promising for monitoring and modeling nutrition, little is known about how food sales log signals could be used for understanding and shaping on-campus nutritional environments.

### 4.2 Opportunities and implications: a call to action

Moving forward, we identify knowledge gaps and outline sketches of themes for analyses to be done at other institutions and other campuses to shape the nutritional environments. We identify five specific areas where we call on the nutrition research community to apply their expertise. Below we propose a research agenda and specify how different research paradigms can answer this call to action to improve nutritional environments.

*(1) Deriving generalizable insights about on-campus dietary behaviors*. Each campus is an independent eco-system, which makes it challenging to derive findings that hold between campuses. Estimates are typically produced at different times and different locations. Furthermore, cultural factors can, in important ways, alter behaviors—for instance, due to varying susceptibility to stress. What behaviors can generalize across campuses around the world? What behaviors are shared between various types of campuses (e.g., educational vs. industrial vs. corporate vs. medical)?

Future efforts might include creating a network of partner institutions that would enable researchers to replicate the same set of analyses and then perform meta-analyses to discover universal behavioral patterns. We envision developing a system to enable processing the anonymized purchase logs (in a pre-determined format) and obtaining and sharing aggregated high-level insights with other campuses. An a priori-designed meta-analysis would then be performed across a cohort of campuses via “megastudies” (52). A unified client-side analysis framework could be built to process the anonymized logs, following templates of studies that can be conducted worldwide in order to answer pre-agreed research questions.

*(2) Collecting more detailed on-campus food offering and consumption data*. A major challenge of the efforts to study on-campus food consumption is the lack of transparency regarding food offer and consumption. On campuses, complex sets of factors beyond the knowledge and reach of individuals who consume food often determine the availability of options, the nature of collected data and its usage, resulting in a lack of transparency regarding food. Moving forward, there is a clear need for more robust and open policies. This is an opportunity for nutrition researchers to influence local stakeholders at their respective institutions in order to reject the lack of transparency regarding food offering and consumption, and advocate for sustainable and resilient supply chains (53).

Since institutions are responsible for the health of everyone on campus, there is a pressing need to collect more data about offered food at institutions and campuses globally. Future efforts should involve advocating for and collecting rich information about the food items and communicating it to the public. This information includes but is not limited to foods' origin, distance of the food source from the campus (i.e., whether it originates from local farms), ingredients, nutrients, calories, nutritional scores, sustainability metrics (e.g., carbon footprint), preparation methods, and food waste statistics.

*(3) Establishing on-campus digital cohorts*. Another potential solution addressing the concern about the incompleteness of purchase logs is setting up digital cohorts where students and staff could share their food consumption and other health-related data for research purposes. In particular, the individuals could share information about their dietary intake, calorie intake, and health outcomes (for instance, through electronic health records or well-being surveys). That would allow studying specific conditions closely linked with dietary habits (e.g., diabetes and heart diseases), and behaviors assumed to be linked with food consumption but not well understood. The latter could include, for instance, information about major life events, daily habits, social media usage and web browsing, menstrual cycle, and mental health. Active data collection efforts are required to answer such ambitious research questions with implications for the health of the general population. Such an effort would be a way to address the incomplete nature of purchase logs by capturing dietary intake and health outcomes more completely.

*(4) Discovering needs and priorities regarding the nutritional environment*. Since campuses worldwide face a lack of unified effort toward health and sustainability, there is a need for a closer examination of structural processes of power at the campuses and a need for a better understanding of factors that slow down current efforts toward health and sustainability. In addition, there is no principled and systematic understanding of what individuals on campuses worldwide want and need, as well as the difficulties and challenges they face. Future efforts should explore experiences and perspectives from marginalized and underrepresented subpopulations including essential campus service staff who consume food but traditionally do not play a role in the decision-making.

To tackle the challenges mentioned above, new qualitative and participatory approaches are needed. A major lesson learned through our case studies is that the researcher analyzing the logs cannot assume the role of a friendly outsider. The researcher needs to talk to stakeholders, understand the events and schedule of the campus, and engage with both food providers and consumers. In our study, we benefited from having the respective information by being embedded into campus. Similarly, there is a need to acknowledge that food offering, the collected data, and the derived insights embody and reproduce the values of those who designed the food offering systems and data collection mechanisms, to begin with (54). However, the values of individuals who are part of the campus should be discovered, not assumed (55), while enabling individuals on campuses to identify their own priorities and make decisions about the food system and about the future use of digital traces they contribute. In the process, the goal is to give a voice to all stakeholders and enable everyone involved to answer questions such as “How is our data being collected? Will the findings be of benefit to us?” (56).

*(5) Developing new principles and practices around ethics and privacy*. Since behavioral data can be misused and purchases can reveal potentially sensitive information about individuals (57), it is necessary to balance the potential to do good with the potential to harm. At present, corresponding institutional approvals are needed to perform analyses within one campus, guiding researchers through ethical and privacy concerns. However, new challenges emerge when there is a need to replicate the same analyses across several campuses so as to derive generalizable insights about dietary behaviors. Having all the data, which is potentially sensitive, at a single central point is a risk and a liability.

Future efforts can involve designing an application that would allow locally running purchase log analysis scripts with embedded privacy mechanisms. If there is an agreement about data formats, universal processing scripts can be run locally. Previously, decentralized data processing across silos has been deployed in settings where silos corresponded to hospitals processing medical datasets (58), consistent with the paradigm of federated learning, a privacy-enhancing technique that allows institutions to keep control of the data. Furthermore, federated learning can be used with other privacy-enhancing technologies, such as differential privacy (DP), which ensures that one cannot infer additional information about the original data from the aggregated results (59, 60). To facilitate collaboration over sensitive data, an alternative approach is to take a sensitive dataset as input and generate a structurally and statistically similar synthetic dataset with strong privacy guarantees (61, 62). Finally, secure multi-party computation (63) and trusted environments (64) enable parties to jointly compute a function over their inputs while keeping those inputs private from each other, or from the main processor of the central server. A key barrier toward that goal is determining whether other institutions are willing to provide data to their own researchers to begin with, and whether researchers could share aggregated insights with researchers at other institutions.

### 4.3 Conclusion

On-campus food offer and consumption broadly affect health, performance, and the environment. We make a case for shaping such nutritional environments by leveraging passively sensed food sales logs, typically available by default. Through case studies of food choice determinants in a large university campus, we demonstrate how analyses of such logs can potentially inform policy-making and argue that nutrition researchers are well-positioned to apply the expertise necessary to contribute to improving food offer and consumption across institutions.

## Data availability statement

The original contributions presented in the study are included in the article/Supplementary material, further inquiries can be directed to the corresponding author.

## Ethics statement

The studies involving humans were approved by EPFL Data Protection Officer (the university-appointed Data Protection Officer monitors compliance with data protection laws, informs and advises the university community of their obligations under the law, and assists with issues related to personal data protection). The studies were conducted in accordance with the local legislation and institutional requirements. Written informed consent for participation was not required from the participants or the participants' legal guardians/next of kin in accordance with the national legislation and institutional requirements.

## Author contributions

KG and RZ contributed to data curation, formal analysis, and writing – original draft. AC, EK, RWh, EH, and RWe contributed to conceptualization and methodology. All authors contributed to writing – review and editing.

## Footnotes

^1^Contrary to traditional data sources—e.g., surveys—digital data is passively collected in order to support regular campus operations, meaning that the act of data collection does not impact or interfere with people's behaviors and the measurements do not rely on self-reporting.

^2^Except for one restaurant, which closed in 2016 and re-opened later in the same year.

^3^The university-appointed Data Protection Officer monitors compliance with data protection laws, informs and advises the university community of their obligations under the law, and assists with issues related to personal data protection.
