# Supplementary material for: Measuring and shaping the nutritional environment via food sales logs: case studies of campus-wide food choice and a call to action
Source: Front Nutr. 2024 Jun 4;11:1231070. doi: 10.3389/fnut.2024.1231070 (PMC11186467; doi:10.3389/fnut.2024.1231070)
Supplement: Supplementary file 1 [file Data_Sheet_1.pdf]

Supplementary Material

1 SUPPLEMENTARY INFORMATION ABOUT THE PURCHASE LOGS DATASET

1.1 Sustainability challenge subpopulation: Demographics

For sustainability challenge participants we leverage demographic information including gender (illustrated in Fig. 1.3: 584 women, 447 men), status at the campus (illustrated in Fig. 1.3: 724 students, 280 staff members, 27 "other"), and birth year (illustrated in Fig. 1.3: average 1991, median 1994, Q1 1988, Q3 1998). In total, 0.6M transactions are attributed with the gender, age, and status of the person who executed it.

Within this subpopulation, the three demographic variables interact. The percentage of women varies by status on campus, and is highest among staff (percentage of women, undergraduate and master students: 56.11%, PhD students: 48.03%, staff: 61.79%). Similarly, average age varies by status on campus (average year of birth, undergraduate and master students: 1997, PhD students: 1991, staff: 1977). Women are also slightly older on average (average year of birth, women: 1890, men: 1992).

Within the subpopulation, we tested the impact of the exam session, separately for students vs. staff. The results are illustrated in Fig. 1.3. As expected, the effect of the exam session on purchasing behaviors is stronger among students, compared to staff.

1.2 Sustainability challenge subpopulation: Representativeness

Fig. S2 illustrates purchases among sustainability challenge subpopulation vs. among the entire population. Besides beer purchases which are more frequent among sustainability challenge participants (likely due to their student status), the sustainability challenge participants tend to execute fewer potentially harmful transactions (fewer energy drinks and products at vending machines and buy more vegetarian meals, both statistically significant with 95% CI). The discrepancy between the general population and the sustainability challenge subpopulation can be explained by both participants self-selecting to participate in the sustainability challenge and participants improving their behaviors due to the participation. Fig. S3 and S4 illustrate the complete distribution of purchases across the sustainability challenge subpopulations.

1.3 Transactions using identifying badge vs. cash or card transactions

We investigated the difference between (1) the entire dataset, (2) non-identifiable cash or card transactions, and (3) identifiable transactions executed with the badge. We identified three systematic differences. Cash or card transactions, compared to transactions executed with the identifying campus badge, are less likely to contain meal purchases (27.63% vs. 35.24%), cost less on average (4.40CHF vs. 4.72CHF), and are less likely to be a vending machine purchase (5.04% vs. 6.70%). The statistics are listed in Table S1.

	% meals	% vending machine	Price (CHF)
Entire dataset	31.67%	5.92%	$M = 4.57$, $STD = 4.45$
Non-identifiable transactions (46.92%)	27.63%	5.04%	$M = 4.41$, $STD = 4.52$
Identifiable transactions (53.08%)	35.24%	6.70%	$M = 4.72$, $STD = 4.39$

Table S1. Dataset distribution across transactions executed using cash or card vs. transactions using identifying campus badge.

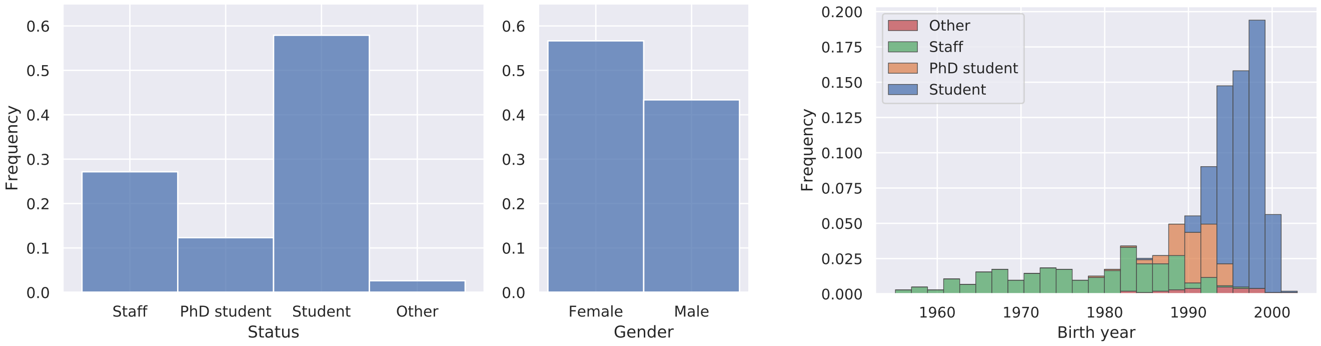

Figure S1. Sustainability challenge participants: demographics. On the left, the histogram of sustainability challenge participants' status (on the left) and gender (on the right). On the right, the histogram of sustainability challenge participants' age by status. "Other" statuses include interns and visitors.

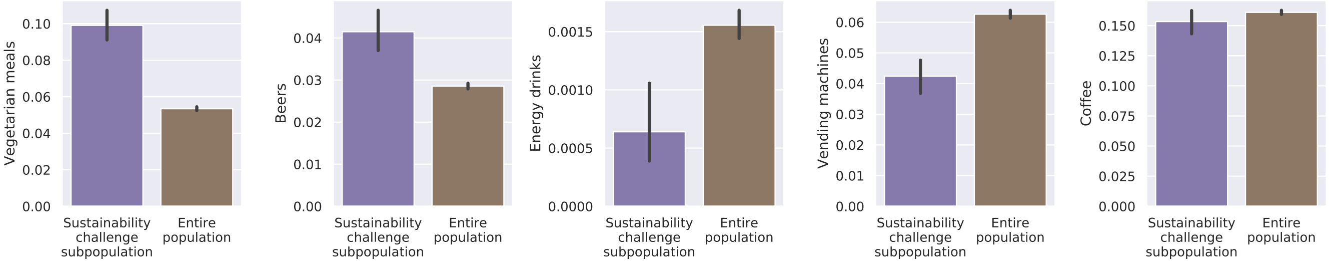

Figure S2. Sustainability challenge subpopulation vs. entire population. Separately for sustainability challenge subpopulation and the entire population (on the x-axis), the fraction of all transactions (on the y-axis) that contain a vegetarian meal, beer, energy drink, vending machine product, or a coffee, averaged across individuals. Error-bars mark bootstrapped 95% confidence intervals. Note the varying y-axis.

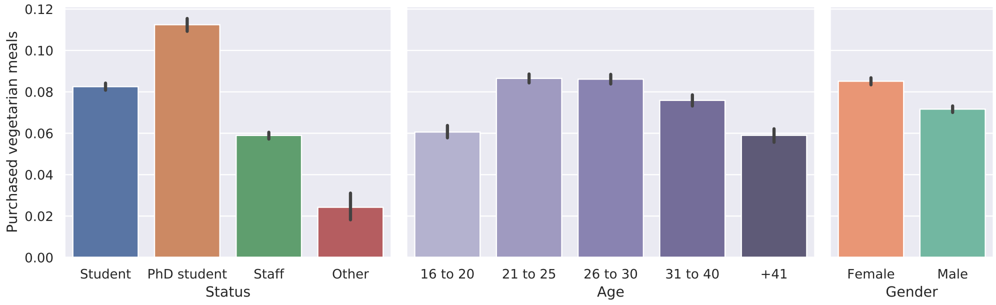

Figure S3. Vegetarian meals across subpopulations. By status, age, and gender strata (on the x-axis), vegetarian purchases (on the y-axis), as the fraction of purchased meals that are vegetarian, among all purchased meals, averaged across individuals. Error-bars mark bootstrapped 95% confidence intervals.

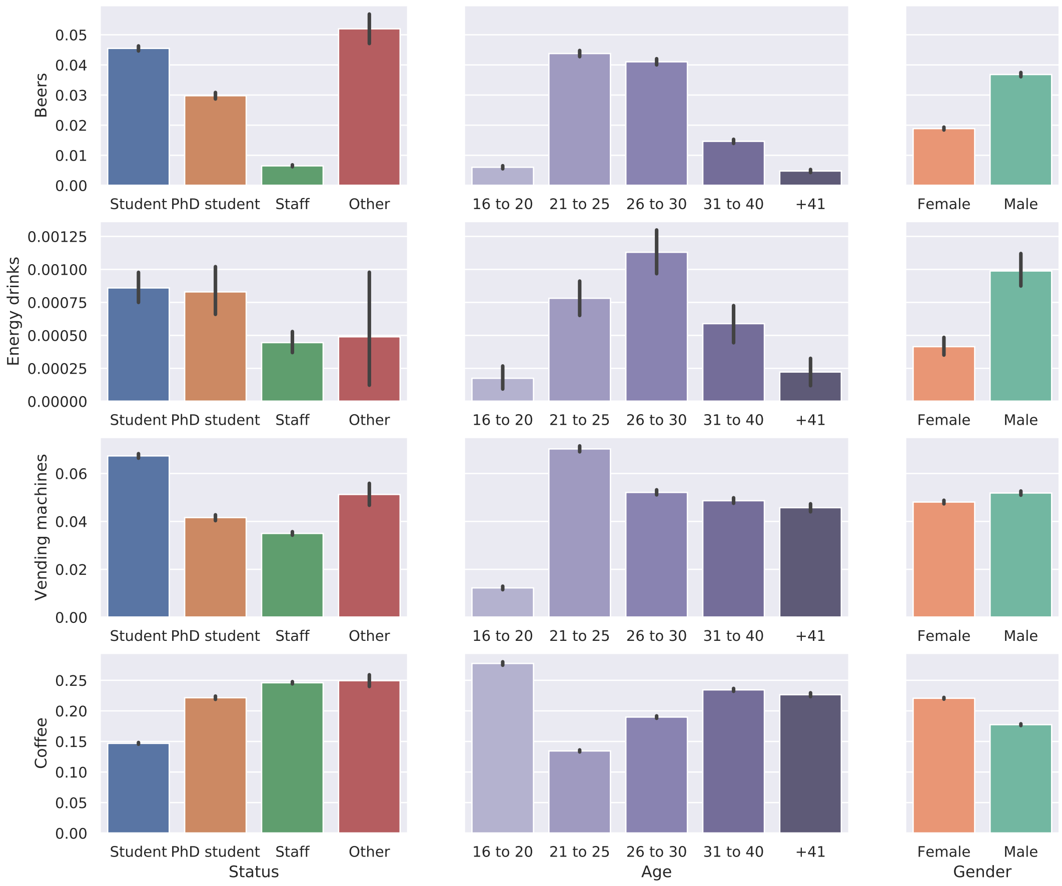

Figure S4. Beers, energy drinks, vending machine, and coffee purchases across subpopulations. By status, age, and gender strata (on the x-axis), the fraction of all transactions (on the y-axis) that contain beer, energy drink, vending machine product, or coffee, averaged across individuals. Error-bars mark bootstrapped 95% confidence intervals. Note the varying y-axis.

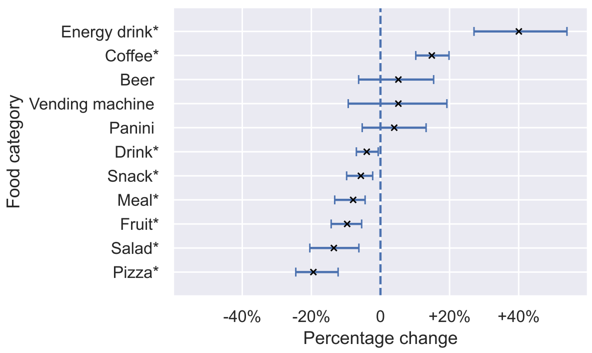

Figure S5. Among students, for different food categories (on the y-axis), the percentage change (on the x-axis) in the fraction of all purchases that contain the food item during exam weeks, compared to lecture weeks.

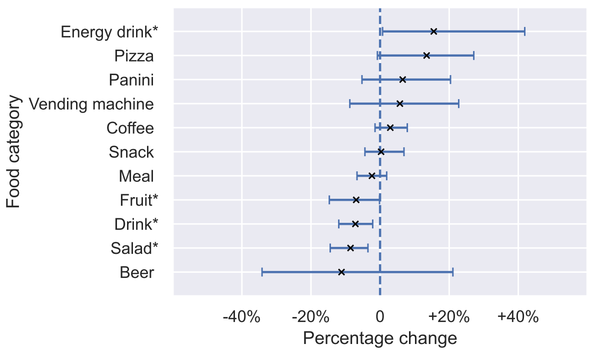

Figure S6. Among staff members, for different food categories (on the y-axis), the percentage change (on the x-axis) in the fraction of all purchases that contain the food item during exam weeks, compared to lecture weeks.
